# The Most Favourable Procedure for the Isolation of Cell-Free DNA from the Plasma of Iso-Immunized RHD-Negative Pregnant Women

**DOI:** 10.5772/62113

**Published:** 2015-12-09

**Authors:** Riyaz Ahmad Rather, Subhas Chandra Saha, Veena Dhawan

**Affiliations:** 1 Department of Obstetrics and Gynaecology, Post Graduate Institute of Medical Education and Research (PGIMER), Chandigarh, India; 2 Department of Experimental Medicine and Biotechnology, Post Graduate Institute of Medical Education and Research (PGIMER), Chandigarh, India

**Keywords:** Cell-free Foetal DNA, Plasma, Iso-immunization, Genome Equivalents, Elution Volume

## Abstract

**Background:**

The ability to achieve quality recovery of cell-free foetal DNA is important for making non-invasive prenatal diagnoses. In this study, we performed quantitative and qualitative analyses of isolated DNA from maternal plasma, using different DNA-isolation methods.

**Method:**

DNA was isolated from 30 iso-immunized women via the QIAamp column-based method, using four different elution volumes and two conventionally based methods. Real-time polymerase chain-reaction quantification of RHD and β-globin genes was performed in order to determine foetal-specific sequences and total genome equivalents, respectively.

**Results:**

The column-based method at a 3 μl elution volume yielded the highest quality and quantity of total DNA (67.0±0.6 ng/μL). At a 3 μl elution volume, the *β-globin* and *RHD*‐gene sequences were estimated to be the highest among all isolation procedures, with 2778.13±1.5 and 66.9±0.6 GEq/mL, respectively, and a 100% sensitivity for *RHD*‐gene sequence detection. Among the two conventional manual methods, the boiling lysis method yielded a higher DNA concentration (53.8±0.8 ng/μL) and purity (1.73±0.05). In addition, the method's sensitivity for foetal-detection sequences was only 80%, whereas the salting-out method's sensitivity was just 70%.

**Conclusions:**

This study confirms the theory that the QIAamp method is a specific and sensitive approach for purifying and quantifying plasma DNA, when used in the minimum elution volume.

## 1. Introduction

Maternal blood contains cell-free foetal DNA (cffDNA), which is detectable as early as the sixth week of gestation; its level rises during the advance of gestational age and disappears rapidly after delivery [1]. Invasive procedures, such as amniocentesis or chorionic villus sampling (CVS), carry the risk of miscarriage as they rely on the sampling of foetal tissues [2]. A consistent, convenient and reliable method has been sought for prenatal diagnosis in order to reduce this risk of miscarriage. The discovery of cffDNA in plasma and serum had a wide application in non-invasive prenatal diagnosis (NIPD), which ultimately reduced the risk of foetal loss by a miscarriage. The many reported applications off cffDNA include RHD genotyping [3][Bibr bibr4-62113]–[5], foetal sexing [6], pregnancy-associated conditions such as pre-eclampsia [7] and aneuploid detection [8]. The quality recuperation of cffDNA in maternal plasma is key for non-invasive prenatal diagnosis; however, limitations in exploring cffDNA for NIPD include the associated recovery of maternal DNA and the failure to recover highly purified cffDNA, both of which impede quantification strategies and assay sensitivity [9]. As cffDNA represents just 3-6% of the total cell-free DNA in maternal plasma, and poses some technical difficulties in DNA extraction due to its extremely low concentrations, an efficient DNA-extraction method or technology is required [10].

To improve the yield of foetal DNA, various methods were designed and formulated for the recovery of plasma from maternal blood and the subsequent DNA isolation, as plasma is considered to be a better source of foetal DNA than maternal serum [11]. For efficient plasma-DNA recovery, multiple methods of centrifugation and filtration of plasma to remove apoptotic bodies and protein impurities have been explored [9]. Commercial kits are used widely to isolate DNA of medium- and large-molecular sizes from body fluids, but less work has been done to isolate and purify fragmented DNA and DNA with a low molecular weight (about 50–150 bp). To improve the quality and concentration of cffDNA, earlier studies have compared manual methods to an automated DNA-isolation system on the basis of sequence-specific copy numbers [12, 13]. Differential elution volumes were used in manual and automated DNA-isolation methods to augment the concentration and foetal-detection sensitivity [14]. Moreover, it was recently proposed that comparison of manual, automated, or commercial DNA-isolation methods increased the recognition, detection and predictability of foetal sequences, because observed concentrations of cffDNA differ depending on the processing and analysis methods used [15, 12]. Due to these challenges, the recovery of an optimal quantity of DNA and foetal-detection markers remain both problematic and exigent. Achieving this goal would pave a path for additional DNA-isolation procedures that can enhance DNA concentration, purity, throughput and efficacy in prenatal testing.

In this study, we have evaluated the suitability of three different methods — one commercial method using different elution volumes and two low-cost conventional methods — by comparing the methods cffDNA yields in terms of concentration, purity and the rate of detection of foetal-specific sequences.

## 2. Materials and Methods

### 2.1 Subjects

The study was conducted in collaboration between the Department of Obstetrics and Gynaecology and the Department of Experimental Medicine and Biotechnology of the Postgraduate Institute of Medical Education and Research (PGIMER), Chandigarh, India. Gestational age was calculated based on menstrual-cycle dates and was confirmed by ultrasound. Where gestational age was not certain, it was confirmed by ultrasound before 15 weeks of gestation had passed. A cohort of 30 RHD-negative iso-immunized women (length of gestation, 23–34 weeks), whose foetus and partner were both RHD-positive, were registered for the study. Pregnancies that experienced intrauterine foetal death, foetal gross-congenital malformations, multiple pregnancies and ones that had an RHD-negative partner were excluded from the study. Full written, informed consent was obtained from all the study subjects prior to their participation. The Institute Ethics Committee of PGIMER approved the study's protocol.

### 2.2 DNA extraction

From each subject, 10 ml of fresh blood was collected from an antecubital vein via an EDTA vacutainer and was transported the same day to the laboratory, where it was kept at −4°C and processed within 12 h. Plasma was separated by centrifuging the samples at 3000 × g for 10 min. Isolated plasma was centrifuged again at 2000 × g for 7 min in order to remove the residual cells and leukocyte carryover. The plasma samples were stored at −80°C before further use. From each sample, 200 μL of plasma was used in order to extract DNA for use in each method. In the commercial column-based method, hereafter referred to as a QIAamp-column method, DNA was extracted from 200 μL of plasma per column, using a QIAamp DNA Blood Mini Kit (Qiagen; Hilden, Germany) according to the “blood and body fluid protocol” [16] suggested by the manufacturer, with a slight adjustment to accommodate the higher plasma volume. The protocol was further modified by incubating samples at 37°C for 2 h instead of 56°C for 10 min. DNA extracted via the commercial column-based QIAamp method was finally eluted in four different elution volumes using an AE elution buffer (50, 30, 10 and 3 μL) in order to check whether or not a modified protocol combined with the use of different elution volumes affects DNA concentration. This method is based on the ability of DNA to bind to the silica surface of the spin-binding column in a chaotropic salt solution after proteinase K lysis has occurred. The second method, hereafter referred to as Conventional Method 1 (CM1), was performed according to the specifications of Miller et al. [17] and involves proteinase K digestion, dehydration and precipitation of proteins using a saturated NaCl solution, followed by phenol:chloroform:isoamyl alcohol extraction and ethanol precipitation. The DNA extracted using this method was eluted in 50 μL of nuclease-free water. The third method involved the lysis of proteins in phenol-based chaotropic salt by boiling samples at 58°C for 10 min, followed by chloroform:isoamyl alcohol and isopropanol precipitation, and 50 μL of nuclease-free water was used to elute the extracted DNA. This method is hereafter called Conventional Method 2 (CM2) [18]. One μL of the undiluted, extracted DNA sample was analyzed using a NanoDrop ND-1000 spectrophotometer (NanoDrop Technologies, Wilmington, DE) in order to check its purity and concentration. The purity of the extracted DNA was based on the A_260_:A_280_ optical density (OD) ratio, and elution buffers used in each protocol were used as controls for OD measures.

### 2.3 Real-time polymerase chain-reaction analysis

Quantitative real-time polymerase chain-reaction analysis (q-PCR) was performed as previously described [19, 20], with modifications to the PCR chemistry and target primers. Eluted DNA from each method was analyzed via a real-time q-PCR ABI 7500 detection system (Applied BioSystems), using power SYBR® Green chemistry. The PCR assay targeted *RHD* loci, a cell-free foetal DNA marker [21] and *β-globin* gene loci. *β-globin* was used as an invariant control and it differentiated between true-negative and false-negative results deriving from a deficient DNA-extraction process. Total DNA and cffDNA quantities were represented as genome equivalents per millilitre (GEq/mL) of plasma, based on copies of *β-globin* and *RHD* sequences detected per microlitre of plasma. The *RHD* primer sequence consists of a forward primer, 5‘-CCT CTC ACT GTT GCC TGC ATT-3’, and a reverse primer, AGT GCC TGC GCG AAC ATT-3‘, resulting in a 74 bp PCR product. The *β-globin* primer sequence consists of a forward primer, 5’-GTG CAC CTG ACT CCT GAG GAG A-3‘, and a reverse primer, 5’-CCT TGA TAC CAA CCT GCC CAG-3‘, resulting in a 102 bp PCR product.

Amplification reactions were set up in a 25 μL reaction volume consisting of a 10 μL power SYBR® Green PCR Master Mix (Applied Biosystems) and 5 μL of template DNA, in addition to primers. Primers were optimized in order to determine the minimum primer concentrations that give the maximum amount of normalized reporter. Primers were used at the final concentration of 300 nM. DNA amplification was carried out in 96 well plates (Applied Biosystems). PCR conditions for all reaction mixtures consisted of an initial denaturation step at 95°C for 10 min and, finally, 40 cycles of 95°C for 15 s, 60°C for 1 min and 72°C for 30 s. Melting-curve analysis was carried out at the end of each PCR assay in order to verify the specificities of the amplified PCR products. An elution buffer was used as a non-template control (NTC) and DNA from a healthy RHD-positive individual was used as a positive control in order to generate a standard curve using 10-fold dilutions (30000, 3000, 300, 30 and 3 GEq/mL) [21]. This standard curve was run in parallel with each PCR reaction. All samples were analyzed in triplicate for both genes, and the mean of the values was determined using the 7700 software and the standard curve of known DNA concentrations [9].

### 2.4 Statistical analysis

A Graph Pad Prism v5.0 (La Jolla, CA, USA) was used for statistical analysis. For each parameter, the mean, the standard error of the mean and the range were calculated. Differences were evaluated via t-tests and non-parametric Mann-Whitney tests, and values of p<0.05 were considered to be statistically significant.

## 3. Results

**Figure 1.**
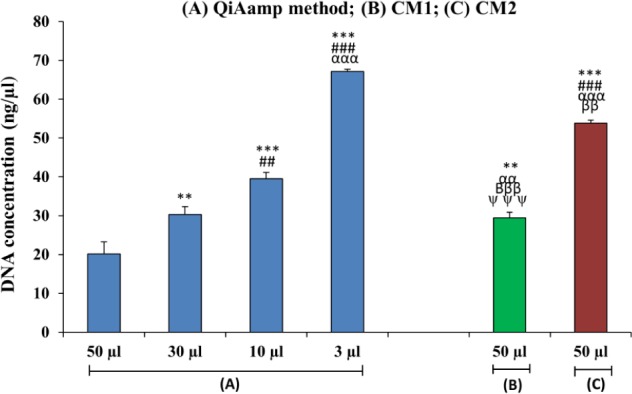
Bar diagram showing a comparison of DNA concentrations isolated from the maternal plasma of iso-immunized women using different isolation procedures. (n=30). (*=50 μL QIAamp method) (#= 30 μL QIAamp method) (α= 10 μL QIAamp method) (β= 3 μL of QIAamp method) (ψ=50 μL CM1); **p<0.01; ***p<0.001; #p<0.05, ###p<0.001; βββp<0.001; αp<0.05, αααp<0.001.

**Figure 2.**
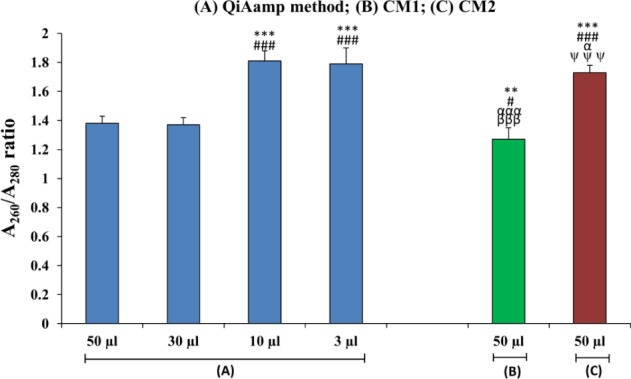
Bar diagram showing a comparison of the quality of DNA isolated from the maternal plasma of iso-immunized women using different isolation procedures. (n=30). (*=50 μL QIAamp method) (#= 30 μL QIAamp method) (α= 10 μL QIAamp method) (β= 3 μL of QIAamp method) (ψ=50 μL CM1); **p<0.01; ***p<0.001; #p<0.05, ###p<0.001; βββp<0.001; αp<0.05, αααp<0.001.

To determine whether a difference in elution volumes using the same method influences DNA's concentration and purity, we checked four different elution volumes in eluting the extracted DNA from plasma using the QIAamp column-based method. As expected, DNA concentrations and purities varied considerably between different elution volumes (Table 1, Figure. 1, 2).

**Table 1.** DNA recovered from maternal plasma of RHD negative iso-immunized women using different DNA isolation methods as measured by spectrophotometer

QiAamp method (n=30)					CM1 (n=30)	CM2 (n=30)
Elution volume	50 μL	30 μL	10 μL	3 μL	50 μL	50 μL
*Mean DNA Concentration (ng/μL)	**20.1±3.1**	**30.3±2**	**39.5±1.6**	**67.0±0.6**	**29.4±1.5**	**53.8±0.8**
Mean DNA purity (A_260_:A_280_ ratio)	**1.38±0.05**	**1.37±0.05**	**1.81±0.07**	**1.82±0.11**	**1.27±0.08**	**1.73±0.05**

* Values are shown as mean with ± standard deviation (SD).

Among the four different elution volumes, the 3 μL volume yielded a significantly higher concentration of DNA (p<0.001) as compared to the three other elution volumes. The DNA concentration derived using the 3 μL volume was 3.36- and 2.26-fold higher than concentrations of the 50 and 30 μL volumes, respectively. However, there was only a 1.6-fold increase in DNA concentration when compared to use of a 10 μL elution volume (p<0.001). These data reveal that there is a negative correlation between the elution volume used and DNA concentration, as an increase in elution volume inhibits high DNA concentration. The overall DNA yield was less while using a 3 μL volume when compared with other volumes using the same method (data not shown). This is probably due to dilution of the sample, which decreases the elution's efficiency. Among the two conventional methods, CM1 yielded a 1.82-fold increase in DNA concentration as compared to CM2 (p<0.001). CM1 proved useful only in obtaining a 1.4-fold increase in DNA concentration when compared with the QIAamp method using a 50 μL elution volume. However, the QIAamp commercial method combined with 10 and 3 μL elution volumes increased the DNA concentration by 1.2- and 2.27-fold, respectively, when compared to CM1, and by 1.3- and 1.2-fold when compared to CM2. These results show that a larger elution volume decreases the overall DNA concentration, and vice versa (Table 1, Figure 1).

The purity of the extracted DNA was counted as a measure of the A_260_:A_280_ OD ratio using a NanoDrop spectrophotometer, and that value is shown in Table 1, Figure 2. The purified DNA sample was expected to show an A_260_:A_280_ ratio of 1.7 to 2.0. In a commercially based QIAamp method, the best ratio was achieved using 10 μL (1.81±0.07) and 3 μL (1.79±0.11) elution volumes; however, this method did not produce optimal ratios when using 50 or 30 μL volumes. Nor did CM1 produce an optimal ratio, suggesting a partial precipitation of proteins reflecting an impure DNA sample. Between the two conventional methods, CM2 showed a significantly improved (p<0.01) A_260_:A_280_ ratio (1.73±0.05) as compared to CM1. Of all methods used, the CM1 procedure was the least efficient DNA purity-yielding method.

The β-globin gene was quantified via q-PCR in order to determine the total GEq/mL, representing the total cell-free DNA (maternal plus foetal DNA). The mean values are shown in Table 2 with ±SD. Among the four different elution volumes used in the QIAamp commercial method, a higher *β-globin* concentration in terms of GEq/mL was achieved (2778.13±1.5) by using a 3 μL volume as compared to a 50 or 30 μL volume, which harvested mean concentrations of 2498.15±1.61 (p<0.001) and 2541.99±4.53 (p<0.001) GEq/mL, respectively (Table 2). This accounts for around 1.11-fold (p<0.01) and 1.09-fold (p<0.05) increases in the *β-globin* concentration compared to use of 50 or 30 μL volumes, respectively. However, no significant increase in the *β-globin* concentration for the 10 μL elution volume was observed when compared with that of the 3 μL volume (p>0.05). In the two conventional methods, CM1 slightly increased the *β-globin* concentration, though to a nonsignificant degree (p>0.05), which is contrary to the spectrophotometric results. This anomaly might be due to the generation of PCR inhibitors, which affects quantification of the *β-globin*.

**Table 2.** DNA recovered from maternal plasma of RHD negative iso-immunized women using different DNA isolation methods as measured by real-time polymerase chain reaction

		QiAamp method (n=30)			CM1 (n=30)	CM2 (n=30)

Elution volume	50 μL	30 μL	10 μL	3 μL	50 μL	50 μL
β-globin (GEq/mL)	2498.15±1.61	2541.99±4.53	2771.8±2.73	2778.13±1.5	1709.4±1.22	1697.5±1.82
Range	(1124.2-3017.8)	(1037.1-3209.1)	(743.0-2817.7)	(798.3-3798.5)	(540.1-4194)	(744.4-2435.8)
RHD (GEq/mL)	49.87±1.4	47.1±0.4	55.2±0.9	66.9±0.6	65.7±1.09	65.1±0.2
Range	(14.3-87.0)	(17.1-79.0)	(19.1-103.6)	(18.6-160)	(27.9-178.12)	(17-172.2)

When a QIAamp commercial method was compared with both conventional methods, it proved to be more efficient and significant (p<0.001) at increasing *β-globin* genome equivalents, for all four elution volumes.

The quantity of cffDNA was determined by estimating the RHD gene sequence for each isolation method (Table 2). Comparison of the two conventional methods, CM1 and CM2, found that they yielded almost equal genome equivalents of *RHD*, with mean values of 65.7±1.09 and 65.1±0.2, respectively (p>0.05). In addition, the *RHD* sequence detection-sensitivity rates were 70% (seven out of 10) and 80% (eight out of 10) for CM1 and CM2, respectively. Among the different elution volumes used in the QIAamp commercial kit, the 3 μL volume yielded the highest concentration (66.9±0.6) of the *RHD* sequences, with 1.3-, 1.4- and 1.2-fold increases in the 50, 30 and 10 μL volumes, respectively. However, the foetal-sequence detection rate was 100% (10 out of 10) for all elution volumes used in the QIAamp column-based method. The concentration of the RHD gene sequences, determined in terms of their genome equivalents, differed consistently when compared with the *β-globin* genome equivalents. When all methods are compared, the QIAamp commercial kit method, used with a 3 μL elution volume, proved the most efficient method for the detection of both *β-globin* concentrations and *RHD* sequences.

## 4. Discussion

The recovery of quality DNA is necessary for proficient molecular diagnostic and clinical investigation [22]. Due to the presence of a small amount of foetal DNA that is associated with complex proteins, the DNA-extraction method needs to be optimized in such a way that the maximum quantity of plasma DNA can be extracted with the fewest impurities [1]. Hence, it is imperative to determine which plasma DNA isolation and recovery methods increase the efficiency, reliability and reproducibility of foetal DNA extraction. To address this quality and quantity issue, we tested three different methods for plasma DNA isolation: the QIAamp commercial method and two conventional methods. The QIAamp commercial method was further tested for the issue of quality DNA recovery, using four different elution volumes. The QIAamp commercial method, when used at a 3 μL elution volume, proved to be the most efficient in terms of overall yield. Using the QIAamp commercial method, we concluded that DNA purity and concentration were at their maximum when using the 3 μL elution volume; however, the detection rates of total and foetal sequences were the same (100%) for all elution volumes, although they differed in achieving optimal DNA concentrations and purities. We did find that using a small elution volume increases the DNA concentration considerably, but also that it correlated negatively with the DNA yield. In the QIAamp method, our study demonstrated a negative correlation between the elution volume, the purity and the quality of DNA and a direct positive correlation between the purity of the samples and the amount of total and foetal sequences detected. These results are in accordance with early studies carried out by Carolina et al. [9]. We achieved overall 3.3- and 1.3-fold increases in DNA concentration and purity, respectively, by using a 3 μL elution volume (Table 1). In addition, total and foetal DNA sequences, measured using real-time PCR, concomitantly increased by 1.1- and 1.3-fold, respectively. These findings suggest that using a smaller elution volume enhances the enrichment of total DNA and small fragments of free foetal DNA. We also observed a 4.8% reduction in Ct value in the q-PCR signal for DNA isolated in a 3 μL elution volume as compared to the three other elution volumes from the same method, which indicates an increased quantity of DNA. This result is supported by other findings showing that DNA eluted while using a smaller amount of elution buffer gives an early Ct value, indicating the presence of concentrated DNA in the sample [23]. Elution carried out with the smallest volume increased the final DNA concentration in the elute, which is very important for any down-streaming process. This result accords with other studies using columns, indicating the ability of the column matrix to enhance DNA concentrations while using a smaller elution volume [24, 25]. However, using smaller elution volumes led to a significantly lower DNA yield. The probable cause for this result may be that the QIAamp column matrix in the presence of the smallest elution volume might have increased the ionic strength, which promoted the highest DNA adsorption with the column matrix. The yield can be improved significantly by eluting the DNA twice using the same buffer; however, dilution renders lower cffDNA quality/concentration, leading to false-negative PCR results. None of the four elution volumes in the QIAamp commercial method gave false-positive results, suggesting the high specificity of this method for cell-free foetal DNA isolation.

To determine the efficiency and reproducibility of the QIAamp commercial method, we compared it with two other conventional DNA-isolation methods, i.e., CM1 and CM2, which are based on either the salting-out and precipitation of proteins or the lysis of proteins by boiling, respectively. CM2 produced an overall higher DNA yield and quality (Table 1) than CM1. However, the total amount of DNA measured by real-time PCR using the *β-globin* loci in CM2 was lower than that of CM1. Partial protein lysis and NaCl contamination (used for the precipitation of proteins) could be the possible reason for the low purity and small quantity of DNA in CM1; however, a nonsignificant total DNA and foetal DNA sequence were detected in CM2 than in CM1 (Table 2). A possible proposed reason might be the loss of smaller-size cffDNA fragments, which might have developed due to an incremental temperature increase that was set up as 58°C for 10 min.

In summary, six different methods for the isolation of cffDNA from the maternal plasma of RHD-negative iso-immunized women were evaluated. A commercially available QIAamp kit at a lower elution volume provides optimal results for DNA purification with high sensitivity and specificity. The resulting high-quality DNA should facilitate precise quantification and sequence analysis, and enable more efficient examination into the molecular nature of the cffDNA in maternal plasma.

## 5. Conflict of interest

The authors declare no conflict of interest.
